# Supramolecular synergy in the boundary lubrication of synovial joints

**DOI:** 10.1038/ncomms7497

**Published:** 2015-03-10

**Authors:** Jasmine Seror, Linyi Zhu, Ronit Goldberg, Anthony J. Day, Jacob Klein

**Affiliations:** 1 Department of Materials and Interfaces, Weizmann Institute of Science, Rehovot 76100, Israel; 2 Institute of Chemistry, Chinese Academy of Sciences, Beijing 100190, China; 3 Wellcome Trust Center for Cell-Matrix Research, Faculty of Life Sciences, University of Manchester, Manchester M13 9PT, UK

## Abstract

Hyaluronan, lubricin and phospholipids, molecules ubiquitous in synovial joints, such as hips and knees, have separately been invoked as the lubricants responsible for the remarkable lubrication of articular cartilage; but alone, these molecules cannot explain the extremely low friction at the high pressures of such joints. We find that surface-anchored hyaluronan molecules complex synergistically with phosphatidylcholine lipids present in joints to form a boundary lubricating layer, which, with coefficient of friction *μ*≈0.001 at pressures to over 100 atm, has a frictional behaviour resembling that of articular cartilage in the major joints. Our findings point to a scenario where each of the molecules has a different role but must act together with the others: hyaluronan, anchored at the outer surface of articular cartilage by lubricin molecules, complexes with joint phosphatidylcholines to provide the extreme lubrication of synovial joints via the hydration–lubrication mechanism.

## Abstract (web summary)

Interacting cartilage surfaces in synovial joints experience very low levels of friction, allowing unhindered mechanical motion. Here, the authors propose how a synergistic interaction of hyaluronan, lubricin and phospholipids, molecules that are ubiquitous in joints, may lead to this high level of lubrication.

## Graphical abstract

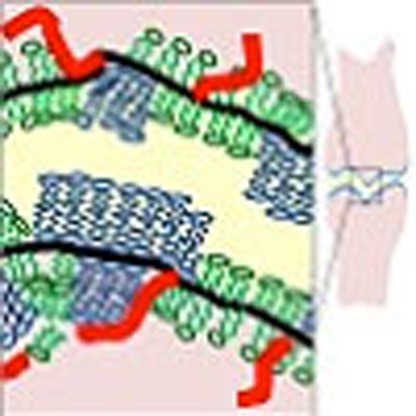

The articular cartilage layers coating the major synovial joints such as hips or knees are remarkable constructs. They not only support a wide range of stresses and impacts^1^,^2^ but, in particular, cartilage surfaces sliding past each other exhibit extremely low levels of friction under physiologically high pressure values (some researchers reporting friction coefficients (μ) down to 0.001 (ref. 3); while pressures to O(100 atm)^1^,^4^ in joints have been reported, including direct measurements with pressure sensors incorporated in implants^1^). This is a lubricity which no man-made surfaces can emulate. A detailed molecular-level understanding of this could have benefits ranging from better treatments of friction-related joint diseases, such as osteoarthritis, to improved devices including prosthetic implants and contact lenses, where low friction is at a premium; but, despite decades of study, such understanding remains elusive^5^,^6^,^7^,^8^. Any insight must, first and foremost, be able to account for the low friction^3^ at the high pressures^9^ of the joints. Hydrodynamic effects have been considered as a lubrication mechanism^7^,^8^,^10^,^11^, but it is likely that a mixed regime including both fluid-film and boundary lubrication operates^12^, and the crucial issue concerns the nature of the boundary layer at the cartilage surface^12^,^13^,^14^. Three quite different components of articular cartilage and of the synovial fluid (SF) surrounding it have each, separately, been invoked as the boundary molecule responsible for the remarkable lubrication of joints: hyaluronan^12^,^15^,^16^,^17^,^18^ (HA), a linear polysaccharide; lubricin^19^,^20^,^21^,^22^, a proteoglycan; and phospholipids^13^,^23^,^24^,^25^,^26^,^27^,^28^. Direct measurements, however^13^,^21^,^22^,^25^,^29^,^30^,^31^, indicate that none of these can, by itself, explain the low friction of the cartilage surface at the high pressures characteristic of the major joints^9^.

In this study we attach HA to a surface—to resemble its configuration at the outer cartilage surface^10^,^21^,^30^,^32^—and find that it complexes with phosphatidylcholines (PCs), ubiquitous in synovial joints^28^, to form robust boundary layers. These layers act synergistically to provide the low friction (μ≈0.001) characteristic of cartilage^3^,^33^, at the highest physiological pressures, and contrast with surface-attached HA on its own, which leads to considerably higher friction. Our results point to a scenario where hyaluronan, phosphatidylcholines and lubricin, each with a very different role, act together to provide the extreme boundary lubrication in articulating joints.

## Results

### Preparation and imaging of surface layers

Freshly cleaved mica sheets were mounted and calibrated in a surface force balance (SFB, see Methods), following which HA was attached via avidin–biotin chemistry, and the surfaces incubated with dipalmitoylphosphatidylcholine (DPPC) introduced into solution in the form of small unilamellar vesicles (SUVs, designated DPPC-SUV) (Methods). The surfaces were then rinsed to remove residual liposome and re-mounted in the SFB, and normal and shear force profiles were measured as a function of load, shear velocity and salt concentration. In extensive controls, we determined interactions between bare mica surfaces, and between HA-coated mica surfaces. We also examined the structure and interactions between initially bare mica surfaces that had been incubated in either a DPPC-SUV solution (no HA) or in a solution containing a bulk DPPC-SUV/HA mixture (no surface-attached HA), followed by rinsing. Freshly cleaved mica surfaces that had been treated identically to those examined in the SFB were imaged using atomic force microscopy (AFM, see Methods).

**Figure 1:**
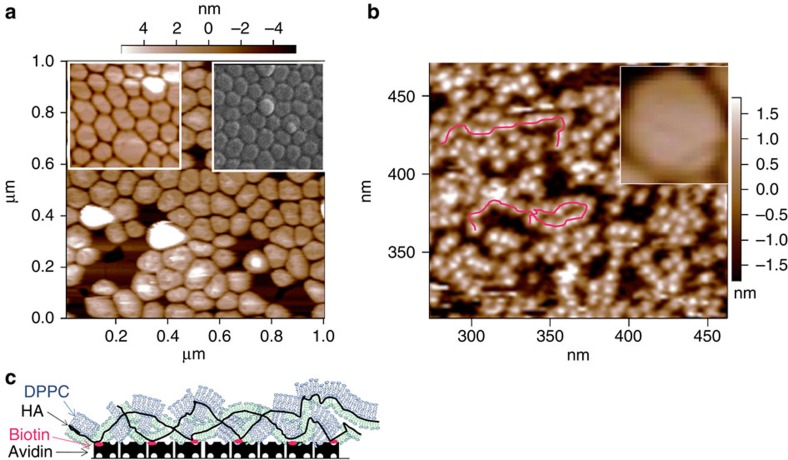
Micrographs of liposomes on mica with and without HA. Tapping mode atomic force microscope (AFM) micrographs of mica surfaces immersed in water following 12±2 h incubation in liposome dispersions and subsequent rinsing (Methods). (**a**) Initially bare mica surfaces following incubation in a DPPC-SUV/HA (dipalmitoylphosphatidylcholine–single unilamellar vesicles/hyaluronic acid) mixture (concentrations 1 mg ml^−1^ of each component) that had been stirred in the dark for 24–48 h at 60–70°C (higher than the liposome solid-ordered to liquid disordered transition temperature *T*_M_(DPPC)=41C). The left inset shows initially bare mica surface following incubation in a DPPC-SUV dispersion (1 mg ml^−1^, no HA) that had been stirred in the dark for 24–48 h at 60–70°C. The right inset shows cryo-scanning electron microscopy image of a mica surface following incubation in a DPPC-SUV dispersion (no HA), from ref. 34 (Reprinted from ref. 34, with permission from Elsevier). (**b**) Mica surfaces coated with avidin and biotinylated HA (bHA) following incubation in a DPPC-SUV dispersion (1 mg ml^−1^, no HA) that had been stirred in the dark for 24–48 h at 60–70°. The inset shows (on the same scale) a single intact liposome taken from the main figure in (**a**). The red lines are a guide to the eye of necklace-like HA-DPPC complexes of structure as attributed in the cartoon (**c**) (blue: bilayers; green: monolayers). These micrographs show that HA in the bulk liposome dispersion has little effect on the liposome attachment to the surfaces (**a**), while when HA is attached to the surface (**b**), it disrupts the liposomes and complexes with the DPPC.

Figure 1a shows initially bare mica surfaces that had been incubated in the DPPC-SUV/HA mixture, followed by rinsing. The intact liposomes are seen to form close-packed layers on the surface, identical to those formed on the mica from a DPPC-SUV solution with no HA (insets to Fig. 1a), indicating that the HA in the bulk solution with the liposomes did little to disrupt their structure or surface attachment. The normal and shear interactions between such close-packed DPPC-SUV layers (attached from the DPPC-SUV/HA mixture) differed little from those between close-packed DPPC liposomes attached from DPPC-SUV dispersions with no HA, studied earlier^34^. In contrast, mica surfaces bearing attached HA chains that had been incubated with DPPC-SUV dispersion, followed by rinsing, as in Fig. 1b, show clearly the disruption of the liposomes and the formation on the surfaces of HA/phospholipid complexes resembling a decorated necklace structure. Inset in Fig. 1b on the same scale, as a contrast, is the image—taken from 1a—of a single intact DPPC-SUV. Since the zwitterionic phosphocholine headgroups of the DPPC are attracted to the negatively charged disaccharide groups of the HA via dipole-charge interaction, it is likely that, when free in solution, the HA adsorbs onto the phosphocholine-exposing vesicles, accommodating to their shape (Fig. 1a). However, when the vesicles interact with HA that is constrained by being attached to a surface and thus cannot accommodate to their shape, the resulting tension leads to the liposome rupture seen in Fig. 1b. From the thickness and lateral dimensions of the necklace-like structures, we may infer that the DPPC lipids form either monolayer elements attached to the hydrophobic moieties on the HA (8 CH_2_ units per disaccharide) by their acyl tails, or bilayer elements attached to the negative-charged polysaccharide via a dipole-charge attraction. In either case we would expect the hydrated phosphocholine headgroups to be exposed, as indicated in the schematic in Fig. 1c.

### Normal surface forces

**Figure 2:**
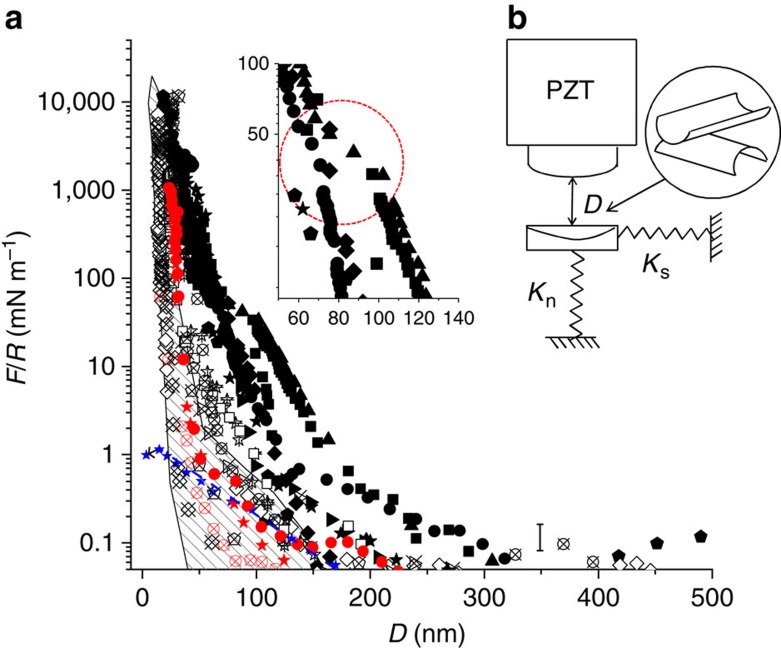
Normal force profiles between avidin-bHA-DPPC-coated mica surfaces. (**a**) Normal forces *F*_n_*(D)* as a function of surface separation D between two avidin-bHA-DPPC-coated mica surfaces as in Fig. 1b, measured in the surface force balance (SFB). Data are normalized as *F*_n_*(D)/R*=2π*E(D)*, where *R* is the mean surface curvature radius and *E(D)* is the interaction energy/unit area. Full symbols are first approaches, crossed symbols are second or third approaches and empty symbols are receding profiles. Black symbols refer to measurements in water, red symbols refer to measurements in 0.15 M KNO_3_ salt solution. A kink often observed in the first approach profiles around *D*≈100 nm (enlarged in inset, circled) is attributed to squeeze-out of residual, loosely attached liposomes. Data are based on five independent experiments with two to four different contact positions in each experiment. (**b**) A schematic of the SFB used for measuring forces between curved surfaces in a crossed-cylinder geometry a closest separation D apart. PZT is the sectored piezo-electric tube providing both normal and lateral motion to the upper surface, while *K*_n_ and *K*_s_ are the springs monitoring the normal and shear forces, respectively (Methods). Error bar, shown at low values of *F*_n_*(D)/R*, corresponds to *δF*_n_*(D)*=±*δD.K*_n_, where *δD*=3 nm is the estimated uncertainty arising from thermal drift and optical fringe errors (Methods). Also shown, for comparison, are the normalized force versus separation profiles between bare mica surfaces across water (controls from present study, blue stars and a broken blue line as guide to the eye), and between mica surfaces coated with DPPC liposomic vectors alone^34^, summarized as a shaded band.

Figure 2 shows the normalized normal force profile *F*_*n*_*(D)/R* versus *D* (surface separation), between two HA/DPPC-bearing surfaces such as shown in Fig. 1b, both in water and in physiological-level salt concentration (0.15 M KNO_3_) where *F*_*n*_ is the normal force and *R* is the mean radius of curvature of the surfaces (Methods). The profiles show an initial long-ranged repulsion, attributed to loosely adsorbed residual liposomes, that had not been effectively rinsed off, on the HA-DPPC complex; these, however, are squeezed out on approach, as indicated by the ‘kink’ in the first-approach profiles (inset to Fig. 2a) and by the shorter range of receding profiles, and of second and subsequent approaches. The limiting surface separation *D*=22±3 nm at the strongest compressions, both in pure water and in salt solution, is consistent with the structure seen in Fig. 1b, attributed, on each surface, to an avidin layer (thickness 4-5 nm), coated with bHA (∼1 nm) complexed with DPPC monolayers or bilayers (∼3–5 nm).

### Friction force measurements

**Figure 3:**
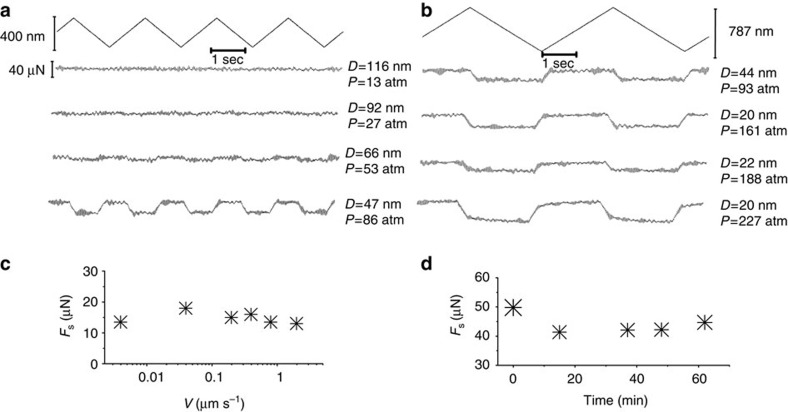
Shear forces measurements. (**a**,**b**) Typical shear force (*F*_s_) versus time traces, taken directly from the SFB, when two avidin-bHA-DPPC-bearing mica surfaces (Fig. 1b) slide past against each other in water. Top zig-zag traces are the back and forth lateral motion applied to the upper mica surface. All the other traces are the shear responses transmitted to the lateral springs at different surface separations and different mean pressures *P*. *P* values (estimated accuracy to ±20%) were evaluated from the contact area *A* derived from the flattening of the interference fringes as *P=F*_n_*/A*. (**c**) shear force as a function of sliding velocity *v*_s_ at pressure *P*=161 atm. (**d**) Shear force as a function of time for a given pressure *P*=61 atm and sliding velocity *v*_s_≈0.4 μm s^−1^. Results reported are based on shear force measurements taken in five different experiments and two to four different contact position within each experiment.

**Figure 4:**
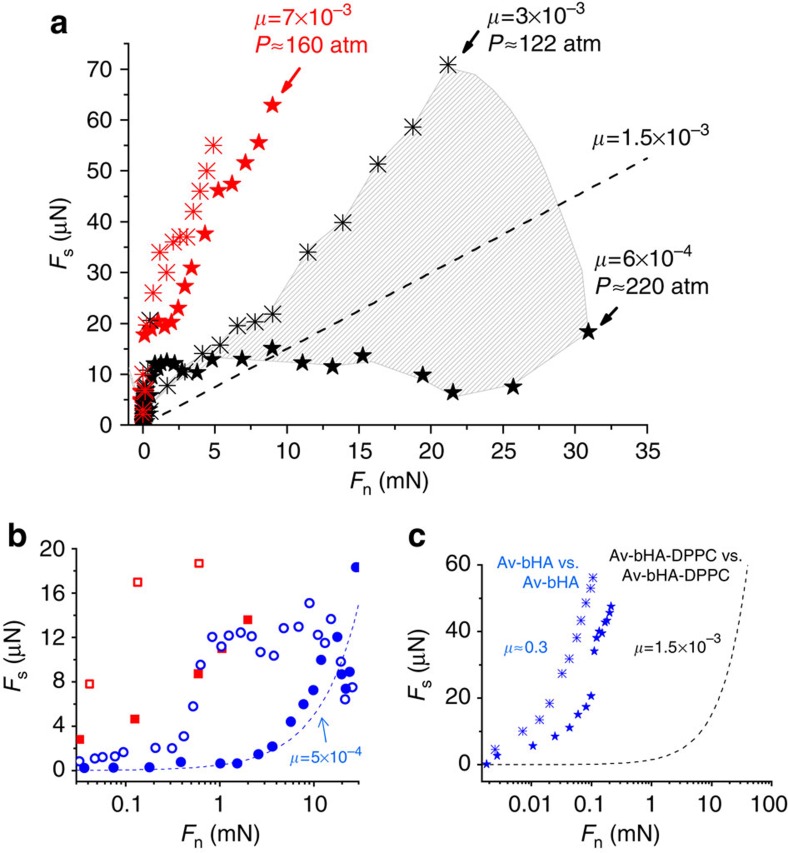
Variation of shear forces with load. (**a**) Variation of shear forces (*F*_s_) with normal load (*F*_n_) between avidin-bHA-DPPC-bearing surfaces across water (black symbols) and 0.15 M KNO_3_ solution (red symbols). The black data points are the *F*_s_ versus *F*_n_ variation for the highest and lowest high-pressure *μ* (coefficient of friction) values, while the shaded area includes all data with intermediate *μ* values (omitted for clarity). The limiting pressures *P* at the maximal loads (*F*_n, max_) for selected profiles and the corresponding values of *μ*=*F*_s, max_*/F*_n, max_ are indicated, while the broken line (*μ*=1.5 × 10^−3^) is a guide to the mean of the data. (**b**) *F*_s_ versus *F*_n_ variation for first (empty symbols) and second (full symbols) approaches at different contact points, showing the reduction in friction following removal of residual liposomes. (**c**) *F*_s_ versus *F*_n_ variation between sliding mica surfaces bearing avidin-bHA alone (before complexation with PC lipids), from this study (stars) and from ref. 30 (crosses); the broken line is taken from the main figure, showing the reduction in *μ* by over two orders of magnitude once PC lipids complex with the HA. Results reported are based on five different experiments and two to four different contact position within each experiment.

Typical friction-force traces between mica surfaces coated with the HA/DPPC complex (Fig. 1b), taken directly from the SFB, are shown in Fig. 3, including variation with sliding velocity (Fig. 3c) and extent of sliding (Fig. 3d). The friction traces reveal low friction coefficients (*μ*≈10^−3^, Fig. 4) up to the highest pressures (> ∼200 atm), while the weak variation with sliding velocity (Fig. 3c) is characteristic of boundary lubrication. The boundary layers are moreover robust, as seen in Fig. 3c where back-and-forth sliding for over an hour at high pressure leads to little change (or even a slight decrease) in the friction force.

Figure 4 summarizes the friction (*F*_s_) versus load (*F*_n_) data, from traces as in Fig. 3, both for first approaches and for second and subsequent approaches of the surfaces. The sharper rise in *F*_s_ at lower loads on first approaches is consistent with dissipation arising from shear of the loosely attached liposomes, before their being squeezed out. The scatter in the data taken from different experiments and different contact points within an experiment may be attributed to heterogeneities arising from different amounts of these residual vesicles at different positions on a first approach. It is of interest that on second and subsequent approaches of the surfaces the initial friction at a given contact point is in general significantly lower, Fig. 4b, as would be expected due to full or partial squeeze-out of the liposomes following the initial approach. The main finding, however, transcending any scatter, is that friction up to high loads and pressures (O(100 atm)) is extremely low, with coefficients in the range *μ*≈1.5±1 × 10^−3^ in water, and around 7 × 10^−3^ in salt solution. These values of the friction coefficient are some two orders of magnitude lower than between the surface-attached HA alone, as seen in Fig. 4c (as discussed further in the following section). Rather similar results (not shown) to those in Figs 1, 2, 3, 4 were obtained when hydrogenated soy PC (HSPC-SUVs) rather than DPPC was used. This is suggestive since HSPC, while not native to cartilage, is a saturated diacyl PC, with predominantly 18:0 (∼ 85%) and 16:0 (∼15%) tails, and such saturated 16:0 and 18:0 tails comprise some 30% of the PCs at the cartilage surface^28^.

## Discussion

These findings shed light on the nature of boundary lubrication in the major synovial joints. Tribometry of unperturbed, *in vivo* articular cartilage is exceedingly challenging, partly because the sliding of cartilage surfaces is so well-lubricated that any measured friction is likely affected by other dissipation pathways (such as distortion of adjacent tissue). In addition, studies on *in vivo* as well as on excised cartilage *in vitro* may be influenced by the known upregulation of cartilage-degrading enzymes within the cartilage in rapid response to insult^35^,^36^. Attempts to understand the extremely efficient boundary lubrication of cartilage have to date thus focused primarily on the molecules that are believed to be the boundary lubricants, most commonly HA^12^,^15^,^16^,^17^,^37^, lubricin^19^,^20^,^21^,^22^ or surface active phospholipids^13^,^23^,^24^,^25^. Any realistic model of cartilage boundary lubrication must, at the very least, be able to reproduce the cardinal features of such lubrication, namely the physiologically low friction coefficient of articular cartilage in joints^3^,^33^ (*μ*≈10^−3^) at the maximal joint pressures^1^,^4^ (O(100 atm) or more), with known components of the synovial joint in their physiological configuration. However, no direct study to date using boundary layers of any of these ingredients (HA, lubricin or surface active phospholipids), either alone or in combination, has managed to do this^13^,^21^,^22^,^25^,^29^,^30^,^31^,^38^,^39^. Thus, in agreement with earlier studies^29^,^30^, we also find high friction (*μ*≈0.3) between mica surfaces bearing HA alone (Fig. 4c), which likely arises from the relatively weak hydration of the HA monomers^30^ (despite the fact that the HA-bearing mica surfaces repel each other across water^30^). Such weak hydration reduces the efficiency of the hydration lubrication mechanism at high compressions (seen also in other polyelectrolytes^40^), resulting in higher energy dissipation, and thus the high friction observed, when the HA monomers rub past each other, as discussed in more detail in ref. 30 (bridging by the HA may also play a role).

In contrast, our present results show that HA that is attached to a surface may complex with PC lipids—such as DPPC—that are present in articular cartilage and in the surrounding SF, to provide a robust boundary layer that fulfills these high-pressure, low-friction requirements. The mechanism underlying the low friction at the sliding interface itself is attributed to the hydration lubrication effect^41^,^42^, arising at the exposed, highly hydrated phosphocholine headgroups of the DPPC coating the surface-anchored HA. In this, hydration layers surrounding charged or zwitterionic groups—including in particular phosphocholine groups—are both tenaciously attached and so can support high pressures, and at the same time are fluid, and so may be sheared with little frictional dissipation. This combination at the slip plane, where the two surfaces slide past each other, underlies the low friction observed. As discussed below (Methods), the remarkable boundary lubrication properties of such surface-attached HA-lipid complexes should also largely apply when they coat mutually compressed cartilage surfaces *in vivo*.

The implications of our findings for boundary lubrication at the surface of healthy cartilage are clear. HA, ubiquitous in articular cartilage, and exposed at its outer surface, complexes with PCs (also ubiquitous in both cartilage and SF^23^,^28^) to form robust boundary layers capable of providing the low friction at the physiologically high pressure of healthy joints demonstrated here. The attachment of the HA at the cartilage surface may be due to entanglements of the long, flexible, linear polysaccharides with the collagen or other microfibrillar network in the superficial zone^10^, or via its known interactions with the lubricin present in this superficial zone^21^,^32^,^43^,^44^,^45^, or likely in combination. In this proposed scenario, all three of the main synovial joint molecular components that have previously been widely conjectured to act independently as boundary lubricants—HA, lubricin and phospholipids—act together, each with a very different role, to provide the boundary lubrication characteristic of healthy synovial joints: lubricin in the superficial zone interacts with and immobilizes HA at the outer cartilage surface, and this surface-attached HA in turn complexes with PCs to form a boundary layer acting via the hydration lubrication mechanism at the exposed phosphocholine groups. This picture naturally accounts for the healing of the boundary layers as they wear, since both HA and PCs (produced by chondrocytes or by synoviocytes) permeate the cartilage and synovial cavity, and would thus be available to replace any HA/PC surface-immobilized complexes that may be removed by friction. The insight provided by these findings into the extremely low-friction boundary layer on articular cartilage may have implications for clinical treatments of osteoarthritic joints (such as intra-articular HA injection). It may also help to better understand the nature of the articulation-related shear-stress which induces upregulation of cartilage-degrading enzymes in emerging osteoarthritis, as suggested in recent murine model studies^36^.

## Methods

### Materials

Water for all solutions, for the SFB experiments and the AFM imaging, was purified with a Barnstead water purification system (Barnstead NANOpure Diamond, resistivity=18.2 MΩ, total organic content (TOC)<1ppb). Ruby Muscovite mica grade 1 supplied by S & J Trading, NY was utilized for the SFB experiments and for the AFM. Avidin from egg white (A9275) was supplied by Sigma Aldrich, Israel. Potassium Nitrate salt (A.R., purity >99.99%) was from Merck; DPPC and HSPC lipids were from Lipoid GmbH; medical-grade HA (0.5 to 1.5 MDa) for the biotinylation was from Genzyme; non-biotynilated HA (1 MDa) was from Lifecore Biomedical; biotin-LC-hydrazide and EDAC were from Pierce and Warriner, Chester, UK.

### Biotinylation of HA

The procedure is described in detail in references^46^,^47^. In brief, 5 mg of HA was dissolved overnight in 0.1 M MES, pH 5.5 at a concentration of 5 mg ml^−1^. To a 1 ml HA solution was added 13 μl of 25 mg ml^−1^ EDAC in 0.1 M MES, pH 5.5 followed by 20 μl of 50 mM biotin-LC-hydrazide in dimethyl sulfoxide, and the sample was mixed by rotation at room temperature overnight. The reaction mixture was dialysed extensively against water and particulate material removed by centrifugation (12,000 × *g* for 1 min). The concentration of the bHA was determined using the metahydroxybiphenyl reaction^48^ relative to standards made from HA dried *in vacuo* over cobalt chloride. The bHA (in 0.02% (w/v) NaAzide) was stored at 4˚C.

### Liposomes preparation

Multilamellar vesicles (MLVs) were prepared by hydrating DPPC or HSPC at 70–75 °C (well above their solid-ordered to liquid-disordered transition temperature *T*_M_(DPPC)=41 °C, *T*_M_(HSPC)=53 °C). MLVs were then downsized to form single-unilamellar vesicles (SUVs), ∼80 nm in diameter, by stepwise extrusion through polycarbonate membranes starting with a 400 nm and ending with 50nm-pore-size membrane, using a Lipex 100 ml extruder system (Northern Lipids, Vancouver, Canada). The SUV liposomes were characterized for size distribution by dynamic light scattering.

### AFM of avidin-bHA-DPPC-coated mica

Freshly cleaved mica was glued on a Petri dish and soaked in 0.01 mg ml^−1^ avidin aqueous solution for about 30 min and then rinsed in water for about 1–2 min. The sample was then covered with 49 μg ml^−1^ bHA solution and kept in a humidity controlled chamber for several hours. After rinsing the sample with excess of water, the Petri dish was filled with 5 ml of water to which 0.2 ml of 15 mM of the PC liposome suspension was added. After overnight adsorption the samples were rinsed in water and scanned with an Asylum MFP3D AFM under water using a Veeco-SNL tip (radius ∼2 nm).

### AFM of mica incubated with HA/PC liposomes mixed in the bulk

HA (1 mg ml^−1^) and 1 mg ml^−1^ DPPC lipids in the form of SUV liposomes (made as above) were stirred together in the dark for 24–48 h at *T*≈60–70 °C (above its *T*_M_) following the protocol of ref. 49. A freshly cleaved mica surface, previously glued on a Petri dish, was covered with the HA-DPPC solution (after cooling to room temperature) and kept overnight in a humidity controlled chamber. The sample was then rinsed with water—paying attention not to expose it to air at any point—and scanned as above. AFM-scanned surface configurations for both cases are identical to those used in the SFB measurements.

### SFB measurement procedure

The surface force balance (SFB) technique and the experimental procedure to measure normal and shear interactions between molecularly smooth sheets of mica at separation D (whose absolute value is measured to ±2-3 Å via multiple-beam interferometry) have been described in detail elsewhere^42^,^50^. A schematic of the SFB is shown in Fig. 2b. In brief, known normal and lateral displacements may be applied to the upper surface via a three-stage system of which the sectored piezoelectric tube PZT is the most sensitive, enabling both normal and lateral motion of the upper surface (at variable lateral velocities *v*_s_). The normal and shear forces transmitted between the two surfaces are directly measured from the bending of the respective springs *K*_n_ and *K*_s_. The intrinsic errors in normal force measurements in the SFB arise from errors in measuring the spring deflections via motion of the interferometric fringes and from thermal drift effects, which are difficult to control for, as considered in detail previously^50^ (see error bar in Fig. 2a). The scatter in the normal force profile data in the present study, as seen in measurements from several repeated experiments and contact points in Fig. 2a (based on 5 independent experiments including 9 different contact points), is larger than this intrinsic uncertainty and arises from the following (as also seen and discussed in earlier studies on mica coated by liposomes alone^34^,^51^). In creating the HA-PC complexes, (see below), differing (small) residual amounts of liposomic PC vectors may be adhered after the washing stage (as also noted in the main text when describing the results of Fig. 4), and these result in different extents of steric repulsion between different contact points, as seen in Fig. 2a.

Boundary lubrication measured in the SFB arises from dissipation at the slip plane between the intimately contacting boundary layers rather than by the underlying mica substrates (which are not themselves in direct contact), and is thus characteristic of the boundary layers *per se*. Soft surfaces (such as cartilage) that are rougher than and whose intrinsic nature may be very different from that of mica deform at the physiological pressures in joints, flattening their asperities, to contact each other intimately over their compressed area, as considered in more detail elsewhere^10^. Boundary friction between such compressed cartilage surfaces, when coated with a lubricant layer, is thus largely also expected to reflect the properties of the boundary lubricant molecules *per se* (as the underlying cartilage surfaces themselves would not be in direct contact).

### Forces between avidin-bHA-DPPC-coated mica

HA was attached to the substrate as follows: following calibration in the SFB at bare-mica/bare-mica contact, the surfaces were soaked in 0.01 mg ml^−1^ avidin aqueous solution for around 30 min and then rinsed in water for 1–2 min. Attachment of the polysaccharide was achieved by interacting lightly biotinylated HA (bHA) with the avidin on the mica via the avidin–biotin interaction (and, partly, via electrostatic interactions between the negative HA and the positive avidin), as described in ref. 47. Normal and shear interactions between the avidin-bearing and, following that, between avidin-HA-bearing surfaces were generally measured as controls to ensure the integrity of the surface layers before introduction of the phospholipids. The detailed protocols for the avidin and bHA attachment, and for the controls, are described in ref. 47; only experiments where contaminant-free attachment of HA on the mica was indicated were carried to the next stage. The HA-coated mica surfaces on their lenses were immersed overnight in 10 ml of pure water into which 400 μl of 15 mM DPPC liposomes solution was added, and then rinsed in 400 ml of pure water and remounted in the SFB as close as possible to their original position. Normal and shear interactions were then measured between the avidin-bHA-DPPC-bearing surfaces. Finally, water was substituted with 0.15 M KNO_3_ solution and normal and shear interactions measured again. The results reported are based on five different experiments and 2–4 different contact position in each experiment. The mean pressure *P* was evaluated as *P*=*F*_n_*/A*, where *F*_n_ is the applied normal force; the contact area *A*=π*a*^2^ or π*ab* where *a* and *b* are principal radii of the circular (*a*=*b*) or elliptical contact area arising from elastic flattening of the glue beneath the mica sheets (measured directly from the flattening of the interference fringes^34^). We estimate an uncertainty of ±(15–20)% in *P* due to uncertainties of order 10% in the measured radii. We work at pressures corresponding to those between cartilage surfaces, rather than at corresponding loads, because the friction depends on the stresses acting on the boundary lubricant molecules (see for example, ref. 40). The resulting friction coefficient is then—for a given pressure—independent of the applied load.

### Forces between HA-DPPC liposomes mixed in the bulk

HA and DPPC liposomes were mixed as described above (see procedure for AFM imaging). Following calibration, the meniscus between the lenses was filled up overnight with the HA-DPPC solution. The SFB boat was then filled with pure water taking care to adequately rinse the surfaces without exposing them to air. Normal and shear interactions were measured in water. Eventually the water was substituted with 0.15 M KNO_3_ to measure normal and shear interactions in salt solution (data not shown).

## Author contributions

J.K. conceived the work. J.S. together with L.Z. carried out and analysed experiments. A.J.D. provided the biotinylated HA. J.S. and J.K. wrote the manuscript. All authors discussed the results and commented on the manuscript.

## Additional information

**How to cite this article:** Seror, J. *et al*. Supramolecular synergy in the boundary lubrication of synovial joints. *Nat. Commun*. 6:6497 doi: 10.1038/ncomms7497 (2015).
